# Applying the Digital Health Social Justice Guide

**DOI:** 10.3389/fdgth.2022.807886

**Published:** 2022-02-28

**Authors:** Caroline A. Figueroa, Hikari Murayama, Priscila Carcamo Amorim, Alison White, Ashley Quiterio, Tiffany Luo, Adrian Aguilera, Angela D. R. Smith, Courtney R. Lyles, Victoria Robinson, Claudia von Vacano

**Affiliations:** ^1^School of Social Welfare, University of California, Berkeley, Berkeley, CA, United States; ^2^D-Lab, University of California, Berkeley, Berkeley, CA, United States; ^3^Energy and Resources Group, University of California, Berkeley, Berkeley, CA, United States; ^4^UCSF Center for Vulnerable Populations in the Division of General Internal Medicine San Francisco, Zuckerberg San Francisco General Hospital, San Francisco, CA, United States; ^5^School of Information, University of Texas at Austin, Austin, TX, United States; ^6^Ethnic Studies, University of California, Berkeley, Berkeley, CA, United States

**Keywords:** social justice, digital health (eHealth), mobile health (mHealth), racism and antiracism, equity, privacy and security

## Abstract

**Introduction:**

Digital health, the use of apps, text-messaging, and online interventions, can revolutionize healthcare and make care more equitable. Currently, digital health interventions are often not designed for those who could benefit most and may have unintended consequences. In this paper, we explain how privacy vulnerabilities and power imbalances, including racism and sexism, continue to influence health app design and research. We provide guidelines for researchers to design, report and evaluate digital health studies to maximize social justice in health.

**Methods:**

From September 2020 to April 2021, we held five discussion and brainstorming sessions with researchers, students, and community partners to develop the guide and the key questions. We additionally conducted an informal literature review, invited experts to review our guide, and identified examples from our own digital health study and other studies.

**Results:**

We identified five overarching topics with key questions and subquestions to guide researchers in designing or evaluating a digital health research study. The overarching topics are: 1. Equitable distribution; 2. Equitable design; 3. Privacy and data return; 4. Stereotype and bias; 5. Structural racism.

**Conclusion:**

We provide a guide with five key topics and questions for social justice digital health research. Encouraging researchers and practitioners to ask these questions will help to spark a transformation in digital health toward more equitable and ethical research. Future work needs to determine if the quality of studies can improve when researchers use this guide.

## Introduction

According to the World Health Organization, digital health strategies–the use of apps, text messaging and online interventions for health–can “promote health, keep the world safe, and serve the vulnerable” (1). Digital health can increase access to health education and management when too few professionals can provide it, or if in-person care is impossible (e.g., during the COVID-19 pandemic) (2, 3). Because mobile devices are pervasive (4), digital health interventions can reach people from all socio-economic backgrounds, with the ability to personalize content by literacy and language. Digital health strategies could revolutionize healthcare by helping people self-manage symptoms of disease, lead healthier lives through engagement in healthy behaviors such as regular physical activity, adequate sleep, and proper nutrition–and connect them to health information and resources. This can result in earlier disease diagnoses, better symptom management, lower costs and more equitable distribution of health resources (5).

However, the rapid growth of digital health apps, remote health provision, and online health information can also raise novel health equity challenges (6). Despite its potential for promoting population health and serving marginalized communities, digital health interventions are often not designed for all who could benefit from them, or may have unintended consequences. For instance, many digital health platforms and studies are designed for patients with high levels of existing digital skills, who only speak English (7). Further, as we will explain in this paper, privacy vulnerabilities and power imbalances that plague the field of medicine, including racism and sexism, influence health app design and research. We will provide guidelines for researchers to design, report and evaluate digital health studies to promote social justice in health.

### What Is Digital Health Social Justice?

The most common understanding of social justice is fairness, especially in how people are treated, what opportunities they have, and how decisions are made. Social justice in *health* is not just the right to be free of disease–it is the right for all to enjoy the highest personally attainable standard of physical health, mental health, and wellbeing (8). We define digital health social justice as the equitable opportunity for everyone to access, use, and benefit from digital health, to achieve their greatest standard of health and wellbeing.

#### Who Is This Guide for and How Do I Use It?

The goal of this study was to develop a guide primarily for health researchers. It may also benefit developers, technology providers and (community) health organizations who work with digital health platforms. Prior to starting a project, writing a grant or paper, or evaluating earlier studies, researchers can answer questions in this framework that are relevant to their work. These questions can help to formulate current or future research questions, determine populations to study, and evaluate their results. Digital health is a broad concept that includes mobile health (e.g., apps and text-messaging), wearable devices and telemedicine (9). Though we discuss digital health broadly, we focus on mobile health.

#### Who Are We?

We are researchers, data scientists, clinicians, and community members funded by a University of California-Berkeley Changemaker Technology Innovation Grant. Through our vast experiences, disciplines, and backgrounds we were brought together by the common goal to give researchers, technology providers, and (community) health organizations the tools to design digital health for social justice.

## Methods

From September 2020 to April 2021, we held five unique discussions and brainstorming sessions with researchers, students, and community partners to receive feedback on the first version of our guide and the key questions (see the Supplementary Materials for the format of these sessions). No individual or protected health data were utilized in this process, and all participation was voluntary. We then sent out the revised key questions to six experts in digital health, data science, social justice, privacy, and education, from March to May 2021 for feedback. We conducted an informal literature review in PubMed and Google scholar, using combinations of keywords (e.g., digital health, social justice, ethics, racism, sexism, biases, discrimination).

We also identified real-world examples throughout from several studies including a mobile health study, the Diabetes and Mental Health Adaptive Notification Tracking and Evaluation (DIAMANTE) study, led by some of the authors (AA and CRL). This study seeks to increase physical activity among English and Spanish-speaking patients with lower income, educational attainment, and/or disability who receive care for depression and diabetes at a public healthcare system in San Francisco (10). In addition, we identified examples from a physical activity chatbot study in low-income English and Spanish speaking women recruited from community health centers led by some of the authors (CAF and AA) (11). The aim of the examples was to connect social justice theory to real-world strategies and methods to increase the accessibility and relevance of digital health platforms to marginalized and underserved groups. Below, we discuss the key questions and corresponding sub questions of our framework.

## Results

### Key Topics and Questions

**Figure 1:**
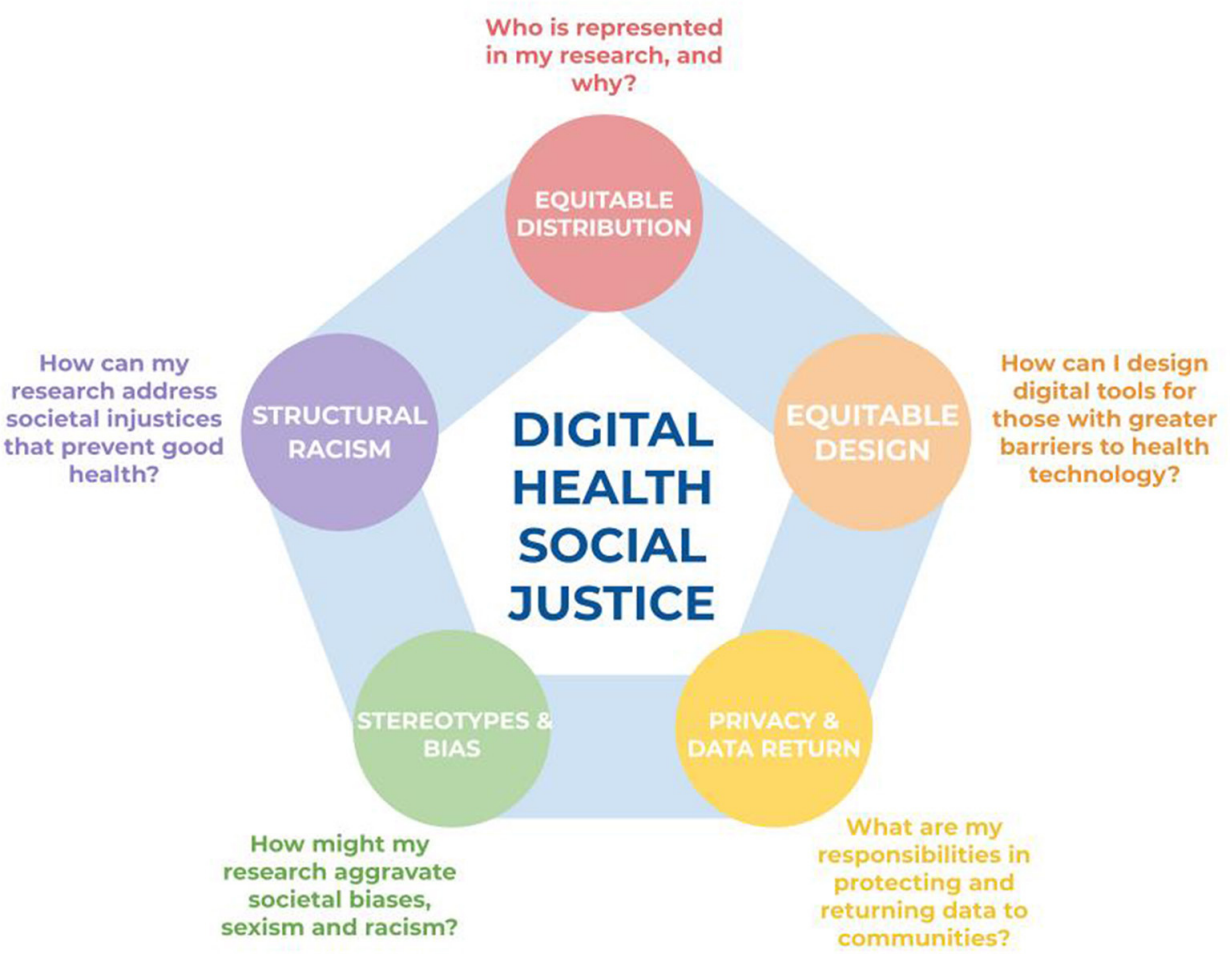
The five key topics and broad questions to guide digital health researchers toward digital health social justice.

We identified five topics, each with an associated key question, that health researchers should ask themselves when designing or evaluating a digital health research study (Figure 1). These topics and questions are:

- Equitable distribution-Who is represented in my research, and why?
- Equitable design-How can I design apps for those with low digital literacy?
- Privacy and data return-What are my responsibilities in protecting and returning data to communities?
- Stereotype and bias-How might my research aggravate societal biases, sexism and racism?
- Structural racism-How can my research address societal injustices that prevent good health?

Below, we described the topics, key-questions and sub-questions in detail. These are also portrayed in Table 1. Table 2 shows the key terms explained in this manuscript. In the Table 3 we show additional resources that can help researchers explore each of the key topics more in-depth.

**Table 1:** Key questions and sub questions.

**Key question**	**Sub questions**
**1. Equitable distribution** *Who is represented in my research, and why?*	• Who (community members/user groups) is going to inform, influence, and be influenced by my research? • What needs, but also resources or strengths do they have that could be built off of? • How might my digital health project have the most value for them? • Are the community members/users with whom I am interacting representative of the population I wish to reach?
**2. Equitable design** *How can I design digital tools for those with* greater barriers to health technologies?	• What does my participants' access to current digital resources (devices, broadband/cellular data) look like? • What is their level of digital, reading, numeracy, and health literacy? • What kind of support, e.g., installing apps, using them daily, do they need to use digital interventions, and how often do they need it?
**3. Privacy and data return** *What are my responsibilities in protecting and returning data to communities?*	• Am I collecting no more data than necessary to answer the research question? • How can I help participants understand the benefits and dangers of participating in the research? • How will data sharing occur throughout the study? • How will I measure the success of my research as a researcher, and more importantly, for my population of interest?
**4. Stereotypes and bias** *How might my research aggravate societal biases, sexism and racism?*	• Am I using “empowering and inclusive” language and design in my app and research? Am I refraining from using terms that may unintentionally harm? • Do my research team and/or (community) partners consist of people from different backgrounds and with different lived experiences? Are everyone's voices heard? • Do I expect differences in outcomes based on participants intersecting identities (e.g., men, women, non-binary individuals, race/ethnicity) within or between groups? How will I analyze this?
**5. Structural racism** *How can my research address societal injustices that prevent good health?*	• To what extent do historical societal injustices affect my (potential) participants' health? • Can digital solutions address these injustices, and how? • Can I examine how these injustices (social determinants of health) affect the success of my interventions?

**Table 2:** Terms and definitions.

**Term**	**Definition**
Digital literacy	The ability to find, use, and compose information through digital platforms
Social justice	Fairness, especially in how people are treated, what opportunities they have, and how decisions are made
Digital health social justice	The equitable opportunity for everyone to access, use, and benefit from digital health, to achieve their greatest standard of health and wellbeing.
Digital health	The use of apps, text-messaging and online interventions for health
Equity	A situation in which resources are distributed and tailored to the needs of the recipients.
Equality	A situation in which everyone has the same resources available to them.
Community-based participatory research (CBPR)	A research approach that involves partnerships between academic institutions, community-based organizations and community participants (10).
Digital intervention	A strategy to decrease delay in receiving help and advice as well as to improve treatment strategies to be evidence-based.
Social justice point of view	Utilizing social justice as the primary lens and objective to analyze the scenario, case, project, or research at hand
Community	A group of individuals that share some commonalities. This can be based on common characteristics (such as place of living, affinity, affiliation, demographics) or by the sheer bond between the individuals.
Social justice framework	A perspective that enables evaluation of a scenario to limit inequity and empowering those who are involved.
Human centered design	An approach consisting of one-on-one interviews, brainstorming sessions, and prototype testing with community members.
Social determinants of health	An individuals' social and/or structural environment, including education, employment and poverty
Open source	Software that is made available to the greater public, usually under a license, that gives anyone the freedom to use, change, and study it.
De-identification of Data	The removal of identifiable information to mitigate privacy breaches.
Algorithm	A process that is usually on the computer that calculates
Informed consent	An agreement with open communication between patient and practitioner for the patient to undergo a medical procedure or participate in a study.
Representation	A study has ample ”representation” in its study participant pool if individuals of different characteristics are sufficiently present in the pool.
Data return	Returning data taken of participants of a study or project to those individuals to benefit and empower them.
Unintended consequences	The materialization of consequences that were not foreseen by researchers or project teams.

**Table 3:** Additional resources to explore the questions outlined by the guide in depth.

**Key question**	**Resources**
**1. Equitable distribution** *Who is represented in my research, and why?*	• Brainstorming session guide: https://www.ideou.com/pages/brainstorming-resources • Field guide to Human Centered Design: https://www.ideo.com/post/design-kit • Inclusive Co-Design Toolkit: http://info.bridgeable.com/inclusive_codesign_toolkit • Experimenting with human centered design: workbook: https://www.tamarackcommunity.ca/library/experimenting-with-human-centered-design
**2. Equitable design** *How can I design digital tools for those with greater barriers to health technologies?*	• Assessing readability: https://www.webfx.com/tools/read-able/ • Measuring digital access and literacy: https://apha.confex.com/apha/2020/meetingapp.cgi/Paper/486628 • Digital literacy training: https://helpathandca.org/digital-literacy/
**3. Privacy and data return** *What are my responsibilities in protecting and returning data to communities?*	• Digital Defense Playbook: https://www.odbproject.org/tools/ • Judgment Call the Game: • AI Blind Spot: https://aiblindspot.media.mit.edu • Videos with privacy notices DIAMANTE study: iPhone (Spanish): https://youtube.com/playlist?list=PLQuqZRju5WHrV0LN_hIC-_9KJOOn6pn5k • iPhone (English): https://youtube.com/playlist?list=PLQuqZRju5WHrJS-K0fNTFPLJFBv8ljYKx
**4. Stereotypes and bias** *How might my research aggravate societal biases, sexism and racism?*	• A toolkit for intersectional gender analysis: https://tdr-intersectional-gender-toolkit.org/cover/0001.html?target=_self&lightbox=0 • Platform for recruiting sexual and gender minority adults into digital health: https://academic.oup.com/jamia/article-abstract/26/8-9/737/5509461 • Inclusive language: https://buffer.com/resources/inclusive-language-tech/~
**5. Structural racism** *How can my research address societal injustices that prevent good health?*	• A Resource to Help Communities Address Social Determinants of Health-CDC: https://www.cdc.gov/nccdphp/dch/programs/healthycommunitiesprogram/tools/pdf/sdoh-workbook.pdf • Resources to explore the ways communities across the country are addressing social determinants of health: https://www.healthypeople.gov/2020/topics-objectives/topic/social-determinants-health/interventions-resources • Framework for assessing the effect of social determinants on behavior change interventions: https://www.tandfonline.com/doi/abs/10.1080/17437199.2020.1718527

### Equitable Distribution

#### Who Is Represented in My Research, and Why?

No one group—gender, racial, ethnic, or socioeconomic—should receive disproportionate benefits or bear disproportionate burdens of research. Technology is often designed to be applicable to the masses in order to gain users and promote the device or platform for profit. This tendency is antithetical to medicine's goal of serving medically specific, often complex subsets of the population. The main users of digital health apps at this time are young, highly educated, tech literate, and free of chronic diseases (12). Most health apps are difficult to use for many. In one study, patients at a public hospital, of which the majority were African-American, had difficulty logging their health data in health apps, understanding the basic functions of these apps, and navigating to the app's main screen (13). Another study found that top-funded, private, US-based digital health companies rarely enroll high-cost, high burden patients with chronic diseases in their studies, or test their impact in terms of outcomes, cost, or access to care (14). Therefore, individuals with complex medical and/or psychosocial conditions may not be the primary digital health audience. All population segments commonly express high interest in digital health platforms (such as electronically communicating with providers or finding online health information), but there are large gaps in groups using digital platforms (15).

Researchers must evaluate where and for whom our interventions can make the most difference. For groups at a greater social disadvantage, such as those who lack access to education, have a low material standard of living, face severe health problems, and whose rights are not protected, the social value of digital health will have greater impact. Particularly for those interested in population-level impact of digital health, equity must be central upfront. The health burden is inextricably tied to social and demographic factors in society and is shaped by historical exclusion, racism, and resource allocation (16). By explicitly focusing on equity within our digital health work, we can better ensure that our interventions have the intended impact to improve population health and reduce disparities rather than exacerbating them. In addition to addressing a justice imperative, ensuring that the most needy are targeted can lead toward a broader social and possibly even economic benefit.

Interventions should be tailored to group's needs (equity), rather than being the same for all (equality). Instead of research starting from the perspective of those in positions of power (the researchers), we need to start from the experience of marginalized individuals. Instead of only focusing on what a community lacks, we need to empower the community to share their knowledge and experiences.

Community-based participatory research (CBPR) is an example of a tool to integrate community knowledge and input. CBPR is a research approach that involves partnerships between academic institutions, community-based organizations and community participants (17). CBPR addresses power imbalances inherent to Western research methods by inviting community members to play an active role in all aspects of the research process, including the design, implementation, and evaluation of interventions. CBPR builds trust and rapport between the community and the researchers, before the intervention begins. CPBR can be combined with human centered design (18), an approach consisting of one-on-one interviews, brainstorming sessions, and prototype testing with community members. Nijagal et al. (18) used a Human Centered Design approach to identify gaps, opportunities, and solutions for perinatal care inequities for Medicaid insured pregnant people in the United States. The authors used the IDEO field guide to Human Centered Design. Methods included semi-structured interviews with stakeholders who received or participated in the care of Medicaid-insured pregnant people, brainstorming sessions to generate prototypes, and community events to test and improve prototypes (see additional resources in Table 2 for the guide). Figueroa et al. (11) describes a virtual co-design session with low-income women for designing a physical activity chatbot. The session was co-led by study participants and included a digital whiteboard where participants could share their needs, wishes and design ideas for a comprehensive health app. Harrington et al. (19) conducted five design workshops to understand the health experiences of low-income African-American older adults living in a residential senior village. These workshops included poster boards to reflect on health needs—using participant's photographs of health related aspects of their environment—visualization, and brainstorming sessions related to health and technology. These studies serve as examples for creating digital tools for health equity. When using design kits or methods from other work, researchers should keep in mind to assess fit with their population of interest, particularly education, digital skills, and literacy level.

### Equitable Design

#### How to Design Digital Tools for Those With Greater Barriers to Health Technologies?

##### Low Digital Literacy, Access, and Trust

Low-income and racial/ethnic minority individuals face greater barriers to health technologies, including digital literacy–the ability to find, use, and compose information through digital platforms–and lack of trust in these technologies (20–22). They less often have smartphones, laptops/computers, and internet connectivity at home (23).

##### Assessing Needs

Many people with low digital skills are interested in using health technology, but have trouble using health apps, because of these structural barriers and poor design (24, 25). Researchers need to assess access to technology (do participants have smartphones, laptops and internet access) and digital literacy during enrollment to determine how much help a participant needs in installing and using apps. Researchers should also ensure that effective non-digital options, such as calling on a regular phone and face-to-face care, are available and accessible (26).

##### Training

Researchers should train individuals with low digital skills to use their health apps at the start, and remain available to provide technical assistance throughout the study. Even if users have access, they may not have the skills to use their smartphone or laptop. We previously developed a framework for assessing digital literacy levels in situations like these (27). Other researchers developed digital skills training to help patients with severe mental illness recognize the need for digital tools, evaluate apps, and use these apps (28). The California Help@Hand project adapted this program to provide community online digital literacy training (see resources). In addition to participant training, researchers should encourage app developers to build in virtual tech support and troubleshooting guides and provide installation support when running research studies. For example, research assistants can help participants download software through phone calls or in-house visits. These types of measures will ensure that those who face high barriers to health technology can participate in digital health research.

##### Design

In the design phase, researchers must make sure the information is accessible for those with low digital literacy, who have often had lower levels of education and have lower reading literacy. Language used in apps should not exceed an 8th grade reading level. Researchers can use tools to check their language level such as WebFX, Grammarly, or the Hemingway App. These tools calculate readability scores, using formulas such as Flesch-Kincaid, based on the length of sentences and words. Researchers can also check with participants if the app's language is understandable and relevant to their needs/wishes in the app development phases. Further, apps should have limited text on the screen and researchers should consider adding audio, voice and video capabilities in addition to text. Adequate design can ensure that participants remain engaged with digital interventions.

*Real Word Example. Prior to the coronavirus pandemic in 2020, the DIAMANTE research team was able to conduct face-to-face research visits with participants. During these visits, researchers were able to help participants understand, install, and download the DIAMANTE app. With the pandemic, we were faced with a major task to conduct this study through online means. This introduced additional obstacles, such as the need for video calling, which most participants were unfamiliar with*.

*We solved this by quickly developing a “digital skills protocol”* (27).

- *From each participant, we mapped out digital profiles of skills and literacy in a phone call*.
- *We divided the participants into two groups: those with sufficient digital skills and those with limited digital skills*.
- *Participants with limited digital skills were then given additional guidance in Zoom calling*.

*One-on-one staff-patient partnerships allowed us to continue our patient recruitment and provide unique technical assistance personalized to each patient's digital profiles. These strategies can mitigate but not eliminate digital barriers for patients without extensive technology experience*.

### Privacy and Data Return

#### What Are My Responsibilities in Protecting and Returning Data to Communities?

The difference in power, where a researcher has control over whether a participant's information is released, should not be taken lightly (29). This is crucial for marginalized communities, who have more often been victims of data abuses. As researchers, we need to increase the participation of marginalized communities by gaining and keeping their trust. To do so, we need to work for and with these communities, involving them in many aspects of the research, including the privacy and dissemination of their data. These should be long-term rather than transactional relationships. Researchers need to inform participants of their rights in study participation, think very carefully about how participants will benefit from their research, and help participants understand the benefits and risks of using health apps. Project funding should include compensation for community member's and organization's time. Finally, community members should also be able to set the research questions and priorities based on their own needs. Kimbrough-Sugick and colleagues proposed that researchers should ask themselves whether their proposed research agenda is driven by external factors such as funding opportunities, personal interests, or institutional priorities, that may not be aligned with the community's agenda (30). To respect the community's values and right to pursue its own interests, researchers should develop relationships with the community (e.g., community leaders, board members, patients, relatives) at an early stage (30). This will help to ensure that research ultimately benefits the health priorities of (marginalized) communities.

#### Privacy Is More Important for Some Than Others

Downloading sensitive apps that collect location, sexual health information, or discuss partner violence comes with greater risks for some. Victims of intimate partner violence, who are more often women of color (31), are vulnerable if abusers can view personal information on their phones, or have installed spyware to track their behavior. Further, location data collection in less densely populated regions increases the risk of breached privacy, putting app users living in rural areas at higher risk (32). Researchers designing sensitive apps can take extra precautions including requiring a password, giving the app an unrelated name and icon, and teaching users how to hide apps from their screens and quickly enable/disable location tracking (33). For example, the Circle of 6 safety app for preventing sexual violence encourages users to select an ambiguous name and uses discreet icons (e.g., a car to represent a need for help getting home; a phone or chat bubble to represent a need for an interruption call on an unsafe date) (34). When we study vulnerable populations or sensitive health issues, we have an even greater responsibility to help individuals protect their phones and their data.

##### Real World Example

*In one of our digital health studies we enrolled a participant with an undocumented immigrant status. We explained to the participant that this app collects location data to track physical activity, but is optional. We wanted to track location to better understand the link between people's travel patterns and their mental health. Because of the participant's status, we advised the participant not to take part in the location tracking part of our study. We helped the participant navigate through the app and indicated where to turn off location tracking. We informed the participant that as long as the settings remain unchanged, location tracking will remain turned off. When the participant needs tech support, a team member is available via SMS or a phone call throughout the duration of the study*.

#### Minimizing Data Collection

The more data we collect, the more potential for harm we create. Researchers and app developers have a responsibility to minimize sensitive information collection, especially when it fails to benefit individuals' health. For instance, a food-tracking app should not collect GPS data if it's unnecessary for the app's functioning. The app's audience often also includes more than the original user. Some mHealth apps request access to microphone or Bluetooth connectivity, which could accidentally collect location data, images or videos of the bystanders (35). Even de-identified data, especially when combining different data streams, can be re-identified. Only a small amount of data is needed to uniquely identify an individual−63% of the population can be uniquely identified by the combination of their gender, date of birth, and zip code (36). Researchers need to handle personally identifiable private information with additional care.

#### Consent Process and Privacy Notices Should Be Accessible

Consent in these studies needs to be fully informed, but privacy notices are often long, tedious, and hard to understand, especially for low digital or reading literacy users. If possible, consent should happen in person. Otherwise, researchers may consider recording video messages (see a link with examples from our team in the resources section). Notices should also be accessible for those with vision (e.g., include large font sizes or auditory notices) or hearing impairments (e.g., include subtitles). Another option is using a standardized color-coded table to give users a quick idea of what information is collected and how it is used or shared. Kelley et al. (37) showed that users achieved higher accuracy of privacy policy knowledge with the standardized tables compared to reading full privacy policies. Thus, consent and privacy notices must be accessible, engaging, and comprehensible for users with low reading literacy.

#### Data Collection Should Benefit the Health of the Researched Community

At the beginning of the study, researchers should plan how they will educate participants post-data collection. Oftentimes, studies benefit the individual researchers in the form of publications or increased grant funding but do not result in tangible benefit to the participants, especially if participants never see the end results of the data collection and analysis. Previous work found that only 27% of clinical trials disseminated results to the participants (38). This is partly because it is often unclear how researchers can turn data into actionable insights (39). Lack of dissemination can impede trust between community partners and researchers.

Some researchers have taken approaches of sending out a newsletter with research progress, offering participants their individual data, or giving them free study materials (such as mobile phone covers with the study logo) to make them feel included in the process. Cunningham-Erves et al. (40) recently developed a stepped framework for community research dissemination, consisting of planning and dissemination phases. There are many open questions including whether it is more informative to return group results and/or individual results, and in what format the results should be returned (articles, blogs, visualizations, or videos). This is an area of research that needs innovation. Nevertheless, researchers must consider and discuss with the community the best way for their study setting to relay the gained knowledge back to participants.

##### Real World Example

*In the DIAMANTE study, our research group started an experiment walking participants through their app data collected throughout the study, and interviewing them about this experience. We inform participants that we are interested in answering questions such as: if people visit certain places, are they also more active? Or, does the number of places they visit relate to their mood? We then show them their most active and least active days, and walk them through the places they visited these days. We inform them that they can always stop sharing location data, or ask us to delete it. We also ask them, if, and why/or why not, they would like to receive their data on a regular basis. This will give us insights into whether participants can learn from their data, see the value in having access to their data, and have greater trust in the research process with increased transparency*.

#### Citizen Science

Another area under debate is citizen-science, scientific research that is conducted in whole or in part by non-professional scientists. The goal of citizen science is to empower non-researchers, crowdsource data, and improve population health. Authors have proposed several frameworks, including combining community based participatory research methods with citizen science, which allows citizens to set the research agenda and have control over their own data (41). Platforms have been developed for data collection from mobile phones, with data remaining on citizen's smartphones for security purposes (for example https://md2k.org/personal). Citizen science is a promising area of research, but recruitment and retention of marginalized populations, protecting data privacy and security, and lack of internet access remain among its challenges (41).

### Stereotype and Bias

#### How Might My Research Aggravate Societal Biases, Sexism, and Racism?

Mobile health, like other health fields, is rooted in biomedicine: a patriarchal system. Medicine has been gendered for centuries and prioritizes certain types of knowledge and practices that create barriers for feminist research and practices (42). Similarly, medicine has a long history of anti-black racism. Women, minority populations, and marginalized gender identities unequally participate in digital health because of this (43). There are gendered differences in app use behavior as well. Men more often use fitness apps and women more often use apps for pregnancy, wellness, and sexual health (44).

Another issue is the biased language and design apps use. For example, apps tend to portray white, thin, young, middle-class, and fertile female bodies as the health standard (45). They place fertility at the heart of sexuality, suggesting that reproduction is central to women's health and that sex is only meant for reproduction (45). Many women do not identify with these images and norms, and may worry that they need to conform to them or will not feel comfortable using such health resources. Digital health has also been slow to include non-binary and genderqueer people (46). Thus, apps reinforce our current, harmful gender norms and promote the idea that digital health spaces are only meant for certain groups (e.g., certain gender identities and ethnicities).

Many digital health efforts may fall short when it comes to health promotion among racial and ethnic minorities (47). This is due in part to a lack of inclusion of racial and ethnic minorities in pilot studies as well as design efforts that don't account for the aforementioned sources of bias. These oversights have produced numerous examples of racial biases built into digital health applications. For instance, a melanoma diagnostic app, trained on images of white skin, more often missed cancerous skin lesions in darker skin (48). Smartwatches have failed to measure Black people's heart rate because the built-in sensors only work on white skin (49).

Researchers and developers must tailor apps to subgroups (such as women, non-binary individuals and minorities) without reinforcing harmful societal norms or stereotypes. Determining what is harmful is challenging, but here we provide some guidelines. Technology providers can pay specific attention to language used within their apps. The Underground Scholars Initiative at Berkeley, a group of formerly incarcerated and system-impacted individuals, developed a guide (50) on using language for communicating about people involved in the carceral system. Researchers also developed guidelines for avoiding racial and gender bias in academic papers (51).

When forming a team for digital health development, team leads must recognize inequities and hire diverse teams (both in terms of social identity and interdisciplinary scholarship). A more diverse group will spot more biases and creative solutions to tackle them. Similarly, including a diverse sample of individuals for piloting and collecting feedback from a digital health product may help bring attention to potential limitations for marginalized groups. Researchers need to pay particular attention to engagement. Because of the barriers minority participants face, they may drop out of digital health research more quickly (52). Minority participants place an increased value on their relationship with the researcher or trial coordinator (53). If participants can imagine a face behind the intervention, understand why they should participate, and feel that the intervention matches their needs they will be more inclined to enroll and remain in the study. Research teams should make an effort to guide participants through this process, and match the cultural and language diversity of participants.

Finally, researchers can examine moderators and mediators to assess for whom and under what social conditions (e.g., gender, race/ethnicity, income, education and their intersections), their digital health interventions are effective (54). Currently, these factors are too often assessed in isolation.

##### Real World Example

*African Americans have a higher risk of cardiovascular disease (CVD) and a 2-fold higher CVD mortality than whites (55). Despite this, there is a lack of suitable interventions for African Americans due to structural racism and social marginalization. FAITH! is an mHealth app based on a church educational program. An interdisciplinary research team including clinicians, technologists, social and behavioral scientists, church leaders and community members designed the app in an iterative process (56). Recommendations from community members during the design phase led to the addition of biblical scriptures and spiritual messages. Because of community involvement and trust building, the study had high recruitment and retention rates, and the app was effective in reducing cardiovascular risk factors in a pilot study (56). This intervention illustrates how researchers can integrate formative and CBPR approaches to design culturally relevant, mHealth lifestyle interventions*.

### Structural Racism

#### How Can My Research Address Societal Injustices That Prevent Good Health?

Researchers need to understand how social injustices influence health, and how their research can contribute to overcoming societal barriers that impede good health. Compared with white individuals, racial and ethnic minorities shoulder a larger burden of many chronic health conditions. For example Latinxs, Black men are twice as likely to die from prostate cancer (56); Black women are 4-times more likely to die from pregnancy related complications (57). Black Americans and Latinxs are 3–4 times more likely to contract, and die from, COVID-19.

Social determinants, including education, housing and employment (58) are the products of structural racism: “the normalization and legitimization of an array of dynamics–historical, cultural, institutional and interpersonal–that routinely advantage white people while producing cumulative and chronic adverse outcomes for people of color” (59). Racial and ethnic minority groups generally have lower education, unequal access to high quality care, and more often live in poverty–these factors all impact health outcomes (59). Chronic ethnic discrimination, acculturation stress, and chronic stress also influence health through various biological pathways, including altered immune system responses (59). Thus, the pathways leading to worse health outcomes for racial and ethnic minorities are multifactorial and multi-domain.

Health apps mostly target individual behavior and rarely pay attention to how social injustices influence health. For instance, mental health apps rarely discuss or acknowledge the role of structural or interpersonal anti-black racism, even though chronic racism is a strong influencer of mental and physical health (60). They also rarely take an individual's social and/or structural environment into account. For example, a mindfulness app might ask individuals to find a quiet space for meditation. While feasible for those living alone, people living in crowded, confined and noisy spaces may struggle to use these interventions. Similarly, an app that practices sleep hygiene, nudging users to find a quiet, dark, and relaxing place with a controlled temperature, fails to support individuals experiencing crowded housing or even homelessness (61).

To acknowledge and address societal injustices, digital health tools should be tailored to participant's environment, resources, and lived experiences. For example, mental health apps could expand beyond symptoms of anxiety and depression, discussing topics such as microaggressions and internalized and structural racism (62). Apps can also specifically target structural issues. For example, an app may connect individuals with support resources within or outside health settings, including (online) women's groups, housing advocacy organizations, food and transportation assistance programs, or education and employment agencies (63). Individuals experiencing homelessness or unstable housing can use mobile apps to participate in electronic case management sessions (64) and to identify and utilize social and health services (65). Researchers should assess how social injustices may affect the success of their interventions, and/or on which level their interventions can address societal injustices.

##### Real World Example

*In a separate physical activity study, we developed a conversational agent (a chatbot) for low-income Latina women* (66). *The app urges them to identify opportunities to walk in the neighborhood. However, our user design research reveals identifying where to walk is a small barrier for exercise. For most participants, childcare is a much larger barrier that stands in the way of regular physical activity. Women reported that they may not always be able to follow our app's suggestions, because they interfere with these responsibilities. An app providing tips about brief exercises using household items that this population can do during the day in between their busy schedules, or activities involving their family, may be more successful*.

## Conclusion

We provide a framework with five key topics and questions to guide researchers wishing to conduct digital health for social justice. The overarching topics we identified were equitable distribution, equitable design, privacy and data return, stereotype and bias, and structural racism. We hope that encouraging researchers and practitioners to ask these questions will help to spark a transformation in digital health toward more equitable and ethical research. Remaining challenges lie in measuring social justice in digital health and comparing this between studies, and in devising strategies to quickly uncover and effectively mediate instances of injustice when they inevitably do happen. Future work needs to determine how to assess if the quality of studies can improve when researchers use this framework. This is a new and developing field. As time progresses, we plan to add to this framework with new insights, knowledge, and feedback. Working toward digital health social justice is crucial for digital health to fulfill its potential of improving health equity.

## Data Availability Statement

The original contributions presented in the study are included in the article/Supplementary Material, further inquiries can be directed to the corresponding author/s.

## Ethics Statement

Ethical review and approval was not required for the study on human participants, in accordance with the local legislation and institutional requirements.

## Author Contributions

CF wrote the first draft of the manuscript. CF, CV, and VR conceptualized the study and the project. CF, HM, PA, AW, and AQ set up the discussion sessions, conducted background literature searches, and contributed to the main body of the text and the guide development. AA and CL were involved in the case studies. AS, AA, and CL helped formulate the key topics and questions. All authors contributed to the writing of the text and approved the final version.

## Funding

This project was supported by a Berkeley Technology Changemaker Grant to CV, VR, and Dr. Harding. CF was supported by the Berkeley Technology Changemaker grant for this project. The DIAMANTE parent trial was funded by an R01 to AA and CL, 1R01 HS25429-01 from the Agency for Healthcare Research and Quality. The physical activity chatbot study was sponsored by the Berkeley Center for Information Technology Research in the Interest of Society and the Center for Technology, Society and Policy.

## Conflict of Interest

The authors declare that the research was conducted in the absence of any commercial or financial relationships that could be construed as a potential conflict of interest.

## Publisher's Note

All claims expressed in this article are solely those of the authors and do not necessarily represent those of their affiliated organizations, or those of the publisher, the editors and the reviewers. Any product that may be evaluated in this article, or claim that may be made by its manufacturer, is not guaranteed or endorsed by the publisher.
