# Supplementary material for: Applying the Digital Health Social Justice Guide
Source: Front Digit Health. 2022 Feb 28;4:807886. doi: 10.3389/fdgth.2022.807886 (PMC8918521; doi:10.3389/fdgth.2022.807886)
Supplement: Supplementary file 1 [file Data_Sheet_1.docx]

**Supplementary material**

**Brainstorming and discussion sessions format**

***Brainstorming session 1 and 2:***

**Digital health Social Justice Worksheet**

**Instructions:** Imagine that you are starting a digital health app study. You can think of the following examples:

1. A mindfulness app that helps people deal with stress and sleep better (for example [calm app](https://apps.apple.com/us/app/calm/id571800810))
2. A physical activity app that helps people exercise more through step tracking and text-messaging (for example [the DIAMANTE app](https://apps.apple.com/us/app/diamante-step-tracking/id1440862542))
3. A diabetes app that captures people’s blood sugars, gives them health advice in real-time and connects them to doctors if needed (for example [welldoc](https://www.welldoc.com/)).

Assign group roles

○ facilitator, rapporteur/note taker, time keeper, cheerleader

Please keep these broad questions in mind while you discuss the set of questions in the break out rooms:

- **Are we asking the right questions, and in the right way (e.g. should we reframe questions)?**
- **Which questions are most important, which may be redundant?**
- **Would these questions work for different fields of study (how could we adapt them)?**

| Break out group 1:  **Who is represented in my research, and why?** | - Am I targeting those who might benefit most from the research I am proposing? - What resources do they already have? Am I duplicating something that already exists? - Which community members/stakeholders should I involve, and how can I help them voice their concerns and needs? |
| --- | --- |

| Break out group 2:  **How might my research aggravate societal biases, sexism and racism?** | - Am I using empowering language and design in my research? - Does my research team consist of a diverse group? Are everyone’s voices heard? - Do I expect differences in outcomes based on subgroups within my study (e.g. men, women, non-binary individuals, race/ethnicity)? Should I analyze this? |
| --- | --- |

| Break out group 3:  **What are my responsibilities in protecting and returning data to communities?** | - How can I explain privacy and security so that participants will understand the benefits and dangers of participating? - How will participants learn about the insights gained from the use of their data? - What data is absolutely necessary to collect to answer the research questions? |
| --- | --- |

***Brainstorming session 3&4:***

**Discussion Questions**

Discuss the following questions with respect to the case study you are examining. If any of the questions are inapplicable, how might you change the study to meet other considerations. Questions in parentheses are to keep in the back of your mind.

Please also note that these questions are mainly to spark conversation-- you don’t have to answer all of these in detail.

Facilitator:

Notetaker:

*1. Who is represented in the research? (Why do you think this group was chosen?)*

*2. What are the potential benefits for participants of this research? (How can my research address societal injustices that prevent good health?)*

*.*

*3. What are the key design elements of this study? (How is the digital component designed for those with low digital literacy?)*

*4. How might the research aggravate societal biases, stereotypes, and discrimination?*

*5. What are the study’s responsibilities in returning data to communities? Does it meet those responsibilities?*

*6. What are the study’s responsibilities in protecting individuals’s privacy? Does it meet those responsibilities?*

**Reflection Questions**

Now that you have a general understanding of the study, what aspects stand out to your group?

*What data & privacy concerns did the researchers consider? What did they not address?*

*As someone not part of this study, what would you change as a researcher?*

*If you were a participant, what would you want in the user agreement about how your data is used? What information would you want to receive about your own health?*

Session 5 was an online presentation of the guide with a question and feedback session.
